# Epidemiology and Genetic Characteristics of Porcine Reproductive and Respiratory Syndrome Virus in the Hunan and Hebei Provinces of China

**DOI:** 10.3390/vetsci10010063

**Published:** 2023-01-16

**Authors:** Wang Zhai, Siyu Yu, Pengxuan Zhang, Yuan Lin, Shenghu Ge, Taojie Zhang, Kun Zhang, Shicheng He, Qiaoyun Hu, Xiaomin Tang, Zhi Peng, Changjian Wang

**Affiliations:** 1 Hunan Provincial Center for Animal Disease Control and Prevention, Changsha 410128, China; 2 College of Veterinary Medicine, Hunan Agricultural University, Changsha 410128, China; 3 Technology Center of Changsha Customs, Changsha 410128, China; 4 Hebei Mingzhu Biotechnology Co., Ltd., Xingtai 055700, China

**Keywords:** porcine reproductive and respiratory syndrome virus, epidemiology, genetic characteristics, ORF5 gene, China

## Abstract

**Simple Summary:**

Porcine reproductive and respiratory syndrome virus (PRRSV) is the major cause of huge economic losses to the pig industry in China. The present study was performed to investigate the epidemiological characteristics of PRRSV in two provinces (Hunan and Hebei) in China in 2021. The results revealed the high prevalence of PRRSV in the healthy and diseased pigs in Hunan and Hebei province. The PRRSV lineages prevalent in Hunan and Hebei province were diverse, while the NADC30-like (sublineage 1.8) and HP-PRRSV (sublineage 8.7) strains predominated. Moreover, four PRRSV strains were isolated in this study, and a novel sublineage 1.8 strain recombinant from the NADC30-like strain and JXA1 (or MLV)-like strain was confirmed via investigating its complete genomic characteristics. In summary, this study has offered the latest information on the epidemiology and genetic characteristics of PRRSV in Hunan and Hebei province, which would be beneficial for PRRS control in the future.

**Abstract:**

Porcine reproductive and respiratory syndrome virus (PRRSV) is a significant threat to the pig industry in China. However, the epidemiological characteristics of PRRSV after the outbreak of African swine fever in China were not thoroughly investigated. In the present study, the serological and epidemiological investigations of PRRSV in pigs from the Hunan and Hebei provinces of China were assessed. The results showed that 73.12% (95% CI 71.74–74.49) of pigs were positive for PRRSV-special antibody by enzyme-linked immunosorbent assay. Out of 5799 samples, 482 (8.31%, 95% CI 7.60–9.02) samples were positive for PRRSV nucleic acids. The positive rates of PRRSV in healthy pigs from farms and slaughterhouses were 2.27% (47/2072) and 7.70% (217/2818), which were lower than that in diseased pigs (23.98%, 218/909). Furthermore, the full-length OFR5 gene sequences of 43 PRRSV strains were sequenced and analysed. Phylogenetic analysis revealed that 43 isolates were classified into three lineages, namely lineage 1 (*n* = 24), lineage 8 (*n* = 15), and lineage 3 (*n* = 4). Lineage 1 could be further divided into sublineage 1.5 (*n* = 2) and sublineage 1.8 (*n* = 22), and lineage 8 was classified into sublineage 8.1 (*n* = 3) and sublineage 8.7 (*n* = 12). Collectively, our findings revealed the severe prevalence of PRRSV in the Hunan and Hebei provinces, where sublineage 1.8 and sublineage 8.7 predominated. The present study provides the update information of the epidemiological and genetic characteristics of PRRSV in the investigated regions, which will be beneficial for PRRS control.

## 1. Introduction

Porcine reproductive and respiratory syndrome virus (PRRSV) is an enveloped, positive-sense, single-stranded RNA virus belonging to the order *Nidoviridales*, family *Arteriviridae* [1]. The disease, porcine reproductive and respiratory syndrome (PRRS), caused by PPRSV, is considered a major viral disease threatening the swine industry worldwide [2,3]. The clinical symptoms of PRRS in pigs are complex, which mainly depend on the infecting lineage, development stage and immune status of the pigs, and environmental conditions [4]. The PRRSV genome is approximately 15 kb in length, consisting of more than ten open reading frames (ORFs) (ORF1a, ORF1b, ORF2a, ORF2b, ORF3, ORF4, ORF5a, ORF5, ORF6, and ORF7) [3]. Of these ORFs, two polyproteins encoded by ORF1a and ORF1b could be further proteolytically processed into 13 non-structural proteins, including Nsp1α, Nsp1β, and Nsp2-Nsp12 [5]. Within the PRRSV genome, the ORF5 encodes the glycoprotein 5 (GP5), which plays important roles in different biological processes, including in virus entry into the target cells and the induction of host immune-response activation [3,6]. In addition, owing to the high genetic variability of ORF5, it is usually used as a molecular marker to investigate the molecular epidemiology and genetic characteristics of PRRSV [3,7].

PRRSV strains are classified into two genotypes (PRRSV-1 and PRRSV-2) and the two genotype strains share nearly 60% nucleotide sequence homology [8]. PRRSV-2 has been widely prevalent in the world, and is further divided into nine lineages based on the genetic features of the ORF5 gene [9,10,11]. Four of the nine PRRSV-2 lineages have been found to be prevalent in Chinese pig herds: lineage 1 (sublineage 1.8, NADC30-like strains and sublineage 1.5, NADC34-like strains), lineage 3 (QYYZ-like strains), lineage 5 (VR2332-like strains), and lineage 8 (sublineage 8.1, Chinese classical PRRSV strains and sublineage 8.7, Chinese highly pathogenic PRRSV strains (HP-PRRSV)) [3,12]. In China, classic PRRSV was the major epidemic-causing strain before 2006. The outbreaks of HP-PRRSV, characterized by high fever and severe reproductive disorders, emerged in 2006, and caused huge economic losses to the Chinese swine industry [13]. Subsequently, the outbreaks of lineage 3 PRRSV strains were first observed in South China in 2010. Another PRRSV lineage, designated as NADC30-like strains, emerged in Chinese pig herds in 2013 [14]. More recently, the emergence of NADC34-like strains has attracted widespread attention in China [15,16]. Furthermore, the co-circulation of multiple lineages of PRRSV in one pig farm has been observed [17].

The epidemiology and genetic variation of PRRSV has been described in some regions or provinces in China [3,7,12], However the relevant data remains limited. In the present study, serum and tissue samples were collected from swine from the Hunan and Hebei provinces in 2021 to investigate the epidemiological characteristics of PRRSV in China. Furthermore, the ORF5 gene sequences of the representative PRRSV strains from different regions were sequenced and analysed. The results showed that PRRSV remained widely prevalent in pig populations in the Hunan and Hebei provinces, with high detection rate in diseased pigs. Meanwhile, multiple PRRSV lineages were circulated in the investigated regions, and the NADC30-like and HP-PRRSV strains were the predominant subtypes. These findings will be beneficial for PRRS control in the future.

## 2. Materials and Methods

### 2.1. Specimen Collection

From March to November in 2021, serum samples from 4007 pigs were collected from different regions nearly covering the whole of Hunan (*n* = 2072) and Hebei (*n* = 1935) provinces, for detection of the PRRSV-specific antibody. These pigs had been previously immunized with commercial PRRSV attenuated or inactivated vaccines. The serum samples were collected from nursing pigs, fattening pigs, sows, and gilts according to the breeding scale [18].

A total of 2818 and 2072 tissue samples, from tonsils or lymph nodes, were obtained from clinically healthy pigs in pig farms and slaughterhouses, respectively. The samples originated from different regions which covered nearly the entire Hunan province. Meanwhile, 359 tissue samples (tonsil, lymph node, and lung samples) were obtained from dead pigs from non-hazardous disposal sites from six regions (Changsha, Yiyang, Yueyang, Huaihua, Xiangxi, and Zhuzhou cities) in Hunan province, and 550 tissue samples (tonsil, lymph node, and lung samples) were collected from diseased pigs with suspected symptoms of PRRS (high fever, reproductive disorders, dyspnoea, etc.,) in Hebei province. 

### 2.2. Enzyme-Linked Immunosorbent Assay (ELISA)

Blood specimens were individually centrifuged at 3000× *g* for 10 min, the supernatants were obtained for detecting anti-PRRSV IgG-specific antibody levels using commercial ELISA kits (IDEXX Laboratories, Westbrook, ME, USA), according to the manufacturer’s instructions. 

### 2.3. Sample Handling and PRRSV Detection 

The total RNA genome was extracted from each collected tissue sample using TRIzol reagent (Cwbio, Beijing, China) following the manufacturer’s introductions. The cDNA was generated from 1 μg RNA using the Revert Aid First Strand cDNA Synthesis Kit (Thermo Fisher Scientific, Waltham, MA, USA), and stored at −20 °C for further processing. Real-time PCR (qPCR) was performed to detect the presence of PRRSV nucleic acids using a commercial PRRSV qPCR detection kit (National Detection Bio, Changsha, China). 

The supernatants of PRRSV-positive samples with low Ct value determined by qPCR were incubated with Marc-145 cells for virus isolation. The virus supernatants were collected for the next-generation sequencing on the Illumina iSeq 100 platform (2 × 150 bp).

### 2.4. Sequencing and Genetic Analysis

The ORF5 gene sequences of 43 PRRSV strains from different regions of Hunan and Hebei provinces were amplified by RT-PCR. The positive PCR products were purified, cloned into the PUCm-T vector (Bioengineering (Shanghai) Co., Ltd., Shanghai, China), and sequenced for further genetic analysis.

Twenty-one reference PRRSV strains were included in this study (Supplementary Table S1). The nucleic acid sequences between the newly obtained PRRSV strains in this study and the reference strains were aligned using DNAstar 7.0 software (Lasergene DNAStar software, Madison, WI, USA) for comparison. Based on the PRRSV ORF5 gene, the phylogenetic tree was reconstructed using the neighbour-joining method in MEGA 7.0 software (Mega Limited, Auckland, New Zealand), with a bootstrap test of 1000 replicates. In addition, the complete genome sequence of four PRRSV strains obtained here were submitted to the GenBank database (GenBank number OP784963-OP784966) and aligned with the reference strains, and the SimPlot version 3.5.1 recombination analysis software (ReduSoft Ltd., Wakefield, UK) was used to identify the putative recombinant events.

### 2.5. Data Analysis

The epidemiological characteristics of PRRSV in pigs with different risks (including region, pig’s development stage, collection site, etc.,) were evaluated using a Chi-square test in SPSS 20.0 software (IBM, Chicago, IL, USA). The difference was considered statistically significant if *p* < 0.05. 

## 3. Results

### 3.1. PRRSV Seroprevalence in Pig Herds in Hunan and Hebei Provinces

From March to November in 2021, 4007 serum samples were collected from different cities or regions in Hunan and Hebei provinces. As shown in Table 1, 2930 serum samples (73.12%, 95% CI 71.74–74.49) were positive for the PRRSV–specific antibody. The seropositive rate of PRRSV in pigs in Hunan province was significantly lower than that in Hebei province (*p* < 0.05). The PRRSV-antibody positive rates in sows and gilts were significantly higher than those in nursing pigs and fattening pigs in the investigated provinces (*p* < 0.05).

### 3.2. Molecular Detection of PRRSV in Clinical Samples

A total of 5799 samples were obtained for detecting PRRSV nucleic acid via qPCR assay. As shown in Table 2, 482 samples (8.31%, 95% CI 7.60–9.02) were positive for PRRSV. The positive rates of PRRSV among samples from pig farms, slaughterhouses, and non-hazardous disposal sites were 2.27% (47/2072), 7.07% (217/2818), and 18.11% (65/359), respectively. which were significantly lower than that in diseased pigs with clinical symptoms of PRRS (27.82%, 95% CI 24.07–31.56).

### 3.3. Phylogenetic Analysis of ORF5 Gene

**Figure 1:**
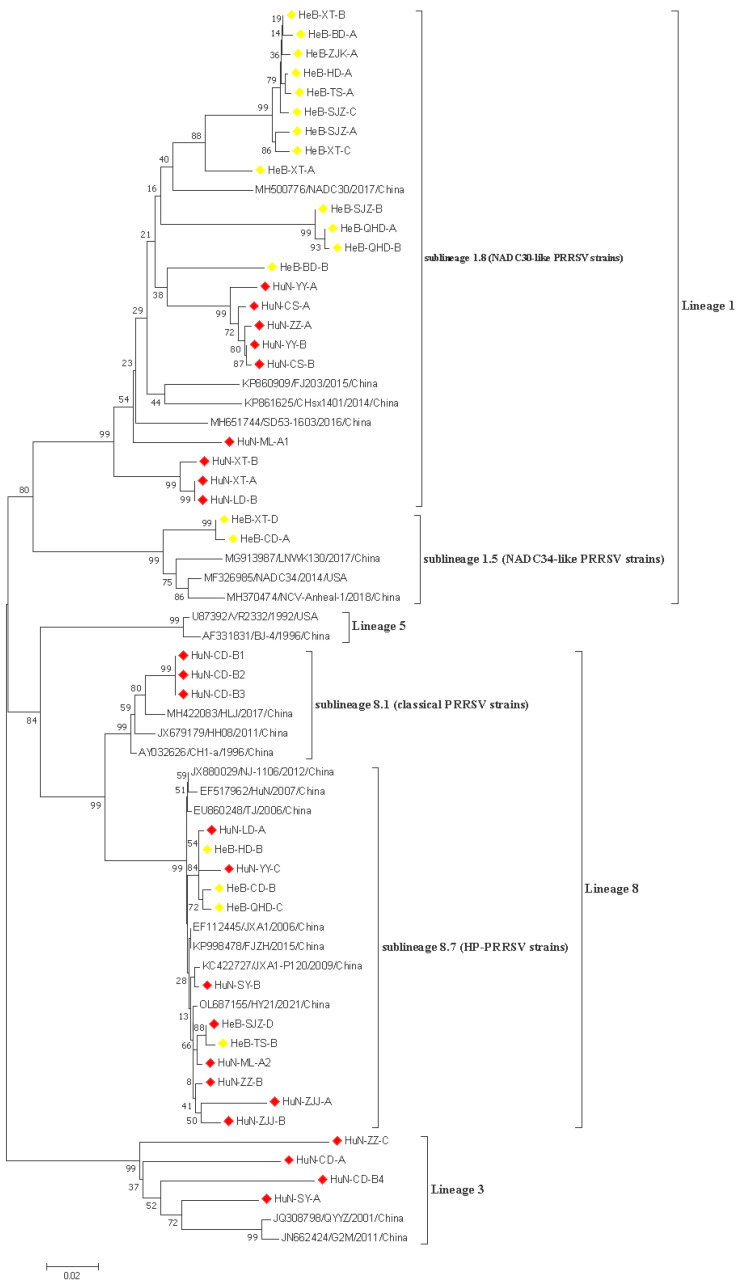
A phylogenetic tree based on the ORF5 nucleotide sequences was generated using the neighbor-joining method with 1000 bootstrap replicates in MEGA X software. Red and yellow prisms represented PRRSV isolates collected from Hunan and Hebei provinces, respectively.

In this study, 43 PRRSV strains were selected to re-construct the phylogenetic tree based on the ORF5 nucleotide sequences. The data showed that all 43 PRRSV strains obtained in this study belonged to the north American genotype, and which were further divided into three lineages: lineage 1, lineage 3, and lineage 8; the proportions of these lineages were 42.85% (24/43), 14.30% (4/43), and 42.85% (15/43), respectively (Figure 1). Three lineages were identified among 23 PRRSV strains obtained in Hunan province, which included NADC30-like strains (lineage 1), HP-PRRSV, and classical PRRSV strains in lineage 8 and lineage 3. Only two lineages were identified among 20 PRRSV strains originating from the Hebei province, which were composed of NADC30-like and NADC34-like PRRSV strains in lineage 1, and HP-PRRSV strains in lineage 8. In addition, the prevalence of multiple lineages was observed in one pig farm. Specifically, the HuN-ML-1 and HuN-ML-2 strains were collected from the same pig farm. The HuN-ML-1 strain belonged to lineage 8.7, while the HuN-ML-2 strain belonged to the lineage 1 (NADC30-like strains).

### 3.4. Sequence Analysis of ORF5 Gene

The full-length ORF5 sequences (600 or 603 bp) of 43 PRRSV isolates were amplified and sequenced (Supplementary Table S1). The ORF5 sequences of the 43 field PRRSV isolates displayed 79.9–100.0% nucleotide identity and 77.6–100.0% amino acid identity. 

Among 43 PRRSV strains identified in this study, the GP5 of 22 strains shared higher sequence homologies with the NADC30 strain compared with the others: 90.5–94.2% and 91.5–94.5% at the nucleotide (nt) and amino acid (aa) level, respectively (Table 3). As shown in the Supplementary Table S2, a series of aa substitutions were observed in the GP5 of 22 lineage 1 PRRSV strains compared with the representative NADC30 PRRSV strain (GenBank number MH500776), such as the positions at sites 13 (Q→P), 15 (P→L), 19 (W→L), 20 (C→Y), 30 (S→N or T), 32–34 (-ND→SNN, MSN, SND, or SNS), 57–59 (NEH→DKR, DKK, GTR, DRH, NKN, NSR, SEK, or GKH). 

12 PRRSV strains showed higher sequence homology with the HP-PRRSV strain (JXA1) (97.0–99.5% and 96.5–99.5% at the nt and aa level, respectively). As displayed in the Supplementary Table S3, 12 aa substitutions were observed in the GP5 compared with the JXA1 strain (GenBank number EF112445), which were mainly located at sites 10 (C→F), 15 (L→P), 23 (F→C or S), 35 (N→S), 58 (Q→R), 59 (K→N or R), 69 (I→L), 104 (G→A or W), 151 (R→K), 164 (G→R), 191 (R→K), 196 (L→R), and 200 (L→P).

A total of 2, 3, and 4 PRRSV strains showed the highest sequence similarities with the NADC34-like, classical, and GM2-like PRRSV reference strains, respectively. A series of aa mutations were also identified in the GP5 compared with the reference strains. Notably, nine aa substitutions in the GP5 in 2 NADC34-like strains were observed when compared with the NADC34 strain (GenBank number MF326985), which were located at sites 4 (K→N), 14 (Q→R), 19 (C→F), 25 (F→L), 29 (V→A), 32 (N→S), 44 (S→G), 98 (A→T), 138 (C→Y). 34 aa mutations in 4 GM2-like strains were found when compared with the GM2 strain (GenBank number JN662424), they were mainly located at sites 6 (S→L), 10 (C→Y), 13 (Q→R), 14 (F→S), 29 (V→A), 59 (T→A/D/G), 60 (N→K), 81 (V→A), 98 (A→T/A), 102 (H→R/Y/C/H), 121 (I→V/T), 151 (K→R), 178 (K→R), and 196 (Q→R). However, 3 classical PRRSV strains identified in this study had only one aa deletion at position site 33 (S) when compared with the CH1-a strain (GenBank number AY032626).

### 3.5. Bioinformatic Analysis of the Complete Genome Sequences of PRRSV Strains

In this study, four PRRSV strains were successfully isolated in Marc-145 cells (Supplementary Figure S1), all of which were collected from Hunan province. Further sequence homology analysis showed that one PRRSV isolate (designed HuN-XT-B, Accession no. OP784963) showed the highest sequence similarity (97.0%) with that of NADC30 strain (Accession no. JN654459). The genome sequences of two isolates (HuN-SY-B, Accession no. OP784965 and HuN-ZZ-B, Accession no. OP784964) shared the highest nucleotide similarities (99.6–99.8% and 99.2–99.6%, respectively) with the JXA1 strain and JXA1-P80 strain (a MLV vaccine strain developed based on the JXA1 strain), and another isolate (HuN-ZJJ-A, Accession no. OP784966) had the highest nucleotide homology (99.8%) with the JSTZ1712-12 strain (Accession no. MK906026).

**Figure 2:**
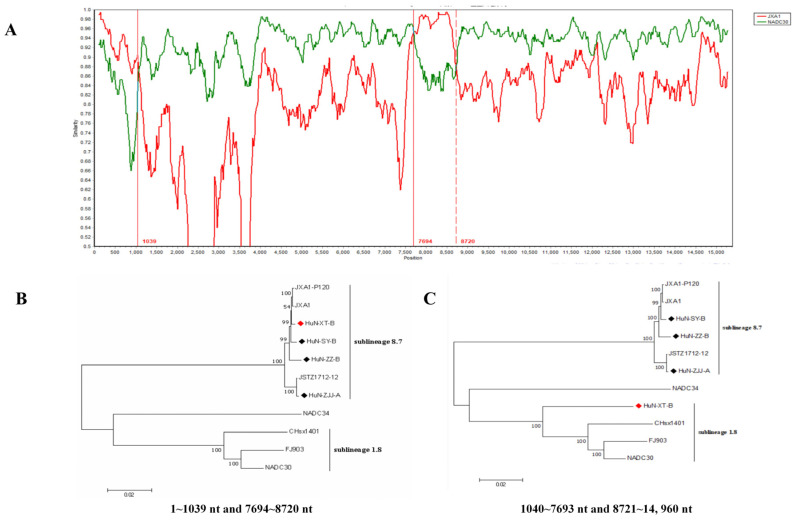
Genome-wide recombinant analysis of PRRSV strain HuN-XT-B predicted by the SimPlot v3.5 software. (**A**) the potential recombination breakpoints of HuN-XT-B were shown as red lines, compared with the parental PRRSV strains (NADC30 and JXA1). (**B**) Phylogenetic tree based on the nucleotide recombination regions (1–1039 nt and 7694–8720 nt). (**C**) Phylogenetic tree based on the nucleotide recombination regions (1040–7693 nt and 8721–14,960 nt). Red prism represented the recombinant PRRSV isolate, black prisms represented the other PRRSV strains isolated in the present study.

Recombinant analysis showed that the recombination events have occurred in the genome of HuN-XT-B strain (Figure 2A), which showed three recombinant breakpoints located in the ORF1a (nt1039) and ORF1b (nt7694 and nt8720) genes. Notably, two regions (1–1039 nt and 7694–8720 nt) were genetically close to the JXA1 strain (highly-pathogenic PRRSV), the other two regions (1040–7693 nt and 8721–14960 nt) showed closer phylogenetic relationship with NADC30-like strains (Figure 2B,C), indicating that the HuN-XT-B strains should be a natural recombinant from the NADC30-like strain and highly pathogenic PRRSV strain. 

## 4. Discussion

The prevalence of HP-PRRSV has been seriously affecting the Chinese swine industry since 2006 [13]. Moreover, owing to the high rate of genetic evolution of PRRSV, the emergence of other PRRSV lineages (such as NADC30-like strains and NADC34-like strains) have also been documented in China in recent years [19,20]. Thus, investigating the epidemiological characteristics of PRRSV would be beneficial for PRRS control. In the present study, 4007 serum samples and 5799 clinical samples were collected from the Hunan and Hebei provinces in 2021 to investigate the prevalence of PRRSV in China. Several epidemiological characteristics of PRRSV were summarized: (1) The results showed that only 73.12% (2930/4007) serum samples were positive for PRRSV antibody. At present, the immunization of swine with PRRS vaccine is not compulsory in China. Meanwhile, owing to the prevalence of African swine fever in China since 2018, the regular immunization of PRRSV vaccines might be ignored in some pig farms. The results indicated that the immunization procedures against PRRS in these pig farms need to be considered or optimized. Only 52.70% (497/943) serum samples from fattening pigs were positive for PRRSV antibody, suggesting that the fattening pigs were at high risk of PRRSV infection. However, the ELISA assay based on detecting the nucleocapsid antibodies cannot distinguish the MLVs-vaccinated pigs and field PRRSV-infected pigs, suggesting that further investigations should be performed to confirm whether the field PRRSV strains are prevalent in these sampled farms. (2) The PRRSV-positive rate among healthy pigs from farms was lower than that of slaughterhouses, being 2.70% (47/2072) and 7.70% (217/2818), respectively. Most of the pigs in slaughterhouses were fattening pigs, and the seropositive rate of PRRSV among fattening pigs was lower than others, which might contribute to this point. However, these data also indicated that etiological examination and antibody monitoring are equally essential for evaluating PRRSV vaccine efficiency, and perhaps even PPRSV eradication. (3) The positive rate of PRRSV among diseased pigs in Hunan and Hebei provinces in 2021 was 23.98% (218/909), which was similar to Guangxi (19.57%, 262/1339) and Zhejiang (22.9%, 450/1963) [3,21], but higher than that in Shandong province (9.58%) [7]. Collectively, the results above revealed the PRRSV has been widely prevalent in China, and the prevalence varied with different regions.

Based on the genetic characteristics of PRRSV ORF5 sequences, PRRSV-2 strains prevalent in China were mainly composed of lineage 1, 3, and 8 [22,23]. Consistently, the phylogenetic analysis showed that all 43 PRRSV strains identified in this study belonged to three lineages (1, 3, and 8). Moreover, the NADC30-like strains become the predominant sublineage, with a proportion of 51.16% (22/43), followed by HP-PRRSV strain (27.91%, 12/43) and lineage 3 strains (9.30%, 4/43), which was in line with the findings of Liang et al. [24]. However, other investigations showed that the HP-PRRSV strain showed the highest proportion among the prevalent strains in some regions [3,25]. Collectively, these results suggested that NADC30-like and HP-PRRSV strains are the major lineages prevalent in China. Notably, the HP-PRRSV, classical PPRSV, NADC30-like, and GM2-like strains were co-prevalent in pig populations in Hunan province, with the proportions of 30.43% (7/23), 13.04% (3/23), 39.13% (9/23), and 17.39% (4/23), respectively. The HP-PRRSV (25.0%, 5/20), NADC30-like (65.0%, 13/20), and NADC34-like (10.0%, 2/20) were co-circulated in the pig industry in Hebei province. These results suggested that the PRRSV lineages prevalent in different regions were diverse. Similar to previous investigations [17,26], multiple PRRSV lineages prevalent in one pig farm were also observed, which might contribute to the occurrence of recombination events in the PRRSV genome. In addition, some of the newly identified HP-PRRSV strains in the present study were genetically close to JXA1 and JXA1-P120 strains, which strongly suggests that biosecurity improvement rather than the application of attenuated vaccines should be the priority for PRRS control [3]. 

The PRRSV GP5 is an important structural protein that participates in various biological processes, including mediating virus entry into host cells, inducing apoptosis, and activating host immune responses [27,28,29]. According to the structure of GP5, three antigenic regions are involved in the activation of host immune response, these being 27–35 aa (early antibody production), 37–45 aa and 52–61 aa (neutralizing antibody production) [2]. Therefore, amino acid substitutions in these regions may alter viral biological characteristics and even contribute to immune escape [30]. In this study, a series of aa mutations were observed in the GP5 region of PRRSV strains (Supplementary Tables S1 and S2). Additionally, some substitution sites were located at the regions of 27–35 aa, such as the sites 35 (N→S) in the lineage 8.7 PRRSV strains, and 26 (A→V), 30 (S→N or T) in the lineage 1.8 PRRSV strains. Further experimental research will be required to investigate the roles of these substitutions in PRRSV strains, such as viral virulence and replication, as well as immunogenicity of the GP5.

Of four PRRSV strains obtained in this study, two PRRSV isolates (HuN-SY-B and HuN-ZZ-B) were genetically closer to JXA1 and JXA1-P80 strains compared with the others, and the JXA1-P80 strain is a commercial MLV vaccine which has been widely applied in the Chinese pig industry for the prevention of PRRS. These observations indicated that the two isolates might be derived from the modified-live virus (MLV), and further investigations will focus on the virulence of these in pigs. In spite of this, the findings suggesting that the biosecurity improvement rather than the application of attenuated vaccines should be the priority for PRRS control, or novel vaccines with high efficiency need be developed for PRRS prevention and control in the future [3]. 

One isolate (HuN-ZJJ-A) showed the highest sequence similarity with JSTZ1712-12 strain which belonged the sublineage 8.7 subgenotype. However, it was reported that the virulence of JSTZ1712-12 strain in piglets was much lower than that of another HP-PRRSV strain (XJ17-5) [23], indicating that the HuN-ZJJ-A strain may have low virulence in piglets, which needs further investigation. 

The NADC30-like strains showed higher recombination probability compared with other lineages, and the current PRRSV vaccines displayed poor cross-protection efficiency against the NADC30-like strains [3]. In this study, the HuN-XT-B isolate was genetically close to NADC30 strain, but they only shared 97.0% nucleotide sequence homologies, combined with the fact that two regions of the HuN-XT-B strain (1–1039 nt and 7694–8720 nt) shared higher sequence similarity with the JXA1 strain, and the other regions of which were highly identical to the NADC30-like strain. The results indicated that the HuN-XT-B strain was recombinant from NADC30-like strain and JXA1 (or MLV)-like strain. Many researchers have reported the occurrences of recombinant events between field strains and HP-PRRSV (or MLV like) strains in China [3,31], suggesting safer vaccines against PRRS should be developed to replace the MLV vaccines which have been widely used in the Chinese pig industry.

In summary, the epidemiology of PRRSV and genetic characteristics of the ORF5 gene of PRRSV strains in the Hunan and Hebei provinces were investigated in this study. The results revealed a relatively low prevalence of PRRSV in healthy pigs, but a high prevalence in diseased pigs in the investigated regions. Multiple PRRSV lineages were prevalent in the Hunan and Hebei Provinces, where the NADC30-like PRRSV strains (sublineage 1.8) and HP-PRRSV strains (sublineage 8.7) were the predominant lineages. Furthermore, we have isolated and reported four PRRSV strains in this study, among them, one novel NADC30-like strain that was recombined from the NADC30-like and HP-PRRSV like strains. These findings have enriched the epidemiology and genetic characteristics of PRRSV and provided the basic data for the prevention and control of PRRS in the investigated regions.

## Figures and Tables

**Table 1:** Seroprevalence of PRRSV among pigs with different development stages in Hunan and Hebei provinces in China.

Regions	Development Stages	Tested Samples	Positive Samples	Positive Rate (%)	95%CI	*p*-Value
Hunan	Gilts	534	413	77.34	73.79–80.89	<0.01
	Sows	591	477	80.71	77.53–83.89	<0.01
	Fattening pigs	436	206	47.25	42.56–51.94	Reference
	Nursing pigs	511	314	61.45	57.23–65.67	<0.01
	Subtotal	2072	1410	68.05	65.34–70.76	-
Hebei	Gilts	421	369	87.64	84.28–91.00	<0.01
	Sows	655	570	87.02	84.26–89.78	<0.01
	Fattening pigs	507	291	57.40	53.09–61.70	Reference
	Nursing pigs	352	290	82.39	78.41–86.37	<0.01
	Subtotal	1935	1520	78.55	76.72–80.37	-
	Total	4007	2930	73.12	71.74–74.49	-

**Table 2:** Molecular detection of PRRSV among tissue samples collected from Hunan and Hebei provinces in China.

Region	Clinical Samples	Samples	Positive Samples	Positive Rate (%)	95%CI	*p*-Value
Hunan	Healthy pigs in slaughterhouses	2818	217	7.70	7.54–7.86	<0.01
	Healthy pigs in farms	2072	47	2.70	2.00–3.40	Reference
	Diseased pigs in non-hazardous disposal sites	359	65	18.11	14.13–22.09	<0.01
Hebei	Diseased pigs with clinical symptoms of PRRS	550	153	27.82	24.07–31.56	<0.01
Total		5799	482	8.31	7.60–9.02	-

**Table 3:** Sequence homology (%) in the ORF5 genes of PRRSV strains obtained in this study and the reference strains.

		Identified Strain					Reference Strain				
		Sublineage 8.1	Sublineage 8.7	Lineage 3	Sublineage 1.8	Sublineage 1.5	VR-2332	CH-1a	JXA1	NADC30	NADC34
Sublineage 8.1 (*n* = 3)	nt	100.0					90.2	97.2	93.5	84.3	86.7
	aa	100.0					92.0	96.5	91.0	83.1	86.5
Sublineage 8.7 (*n* = 12)	nt	91.5–94.2	96.7–99.8				87.2–89.2	92.7–95.2	97.0–99.5	81.5–87.0	86.6–87.7
	aa	89.0–91.5	89.5–98.0				86.1–88.1	89.6–93.0	96.5–99.5	82.1–84.6	86.1–87.1
Lineage 3 (*n* = 4)	nt	83.0–84.8	82.4–84.7	87.6–91.4			81.6–85.9	83.9–85.9	82.9–84.6	79.3–83.5	81.6–85.2
	aa	79.5–82.5	78.6–83.6	88.1–92.5			81.1–84.6	81.5–86.6	80.6–84.1	79.6–84.1	81.6–85.6
Sublineage 1.8 (*n* = 22)	nt	83.2–87.2	81.4–87.1	79.6–84.9	89.2–99.5		83.3–87.1	84.1–88.4	83.3–87.1	90.5–94.2	86.1–92.5
	aa	81.5–85.5	80.6–86.1	77.6–87.1	88.1–99.5		81.6–86.6	82.1–87.1	82.6–87.6	91.5–94.5	86.1–92.5
Sublineage 1.5 (*n* = 2)	nt	85.1–85.4	86.2–87.1	80.8–84.4	86.1–87.9	99.7	81.1–87.7	86.6–86.9	86.6–86.9	84.8–85.5	95.9–96.2
	aa	84.0–85.0	84.6–87.1	79.0–84.1	84.6–89.6	99.0	85.1–86.1	84.6–85.6	85.6–86.6	87.1–88.1	93.5–94.5

## Data Availability

All data generated in the present study was available in the published manuscript and its Supplementary Materials.

## Supplementary Materials

The following supporting information can be downloaded at: https://www.mdpi.com/article/10.3390/vetsci10010063/s1, Figure S1: Viral isolation and the cytopathic effect of representative PRRSV strains in Marc-145 cells. (Note: NC, negative control; HuN-SY-B and HuN-XT-B: two representative PRRSV strains obtained in the present study); Table S1: Detailed information of PRRSV strains identified in this study and reference strains, including strain name, collection year, isolation region, genotype, and GenBank accession numbers; Table S2: Comparison of GP5 amino acid sequences of sublineage 1.8 PRRSV strains identified in the present study; Table S3: Comparison of GP5 amino acid sequences of sublineage 8.7 PRRSV strains identified in the present study.
