# Supplementary material for: Epidemiology and Genetic Characteristics of Porcine Reproductive and Respiratory Syndrome Virus in the Hunan and Hebei Provinces of China
Source: Vet Sci. 2023 Jan 16;10(1):63. doi: 10.3390/vetsci10010063 (PMC9866626; doi:10.3390/vetsci10010063)
Supplement: Supplementary file 1 [file vetsci-10-00063-s001.zip › vetsci-2067089-supplementary.pdf]

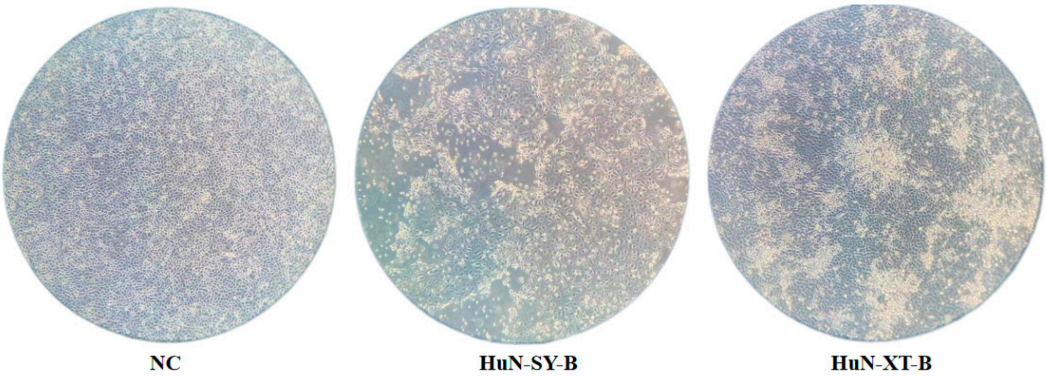

Supplementary Figure S1. Viral isolation and the cytopathic effect of representative PRRSV strains in Marc-145 cells. (Note: NC, negative control; HuN-SY-B and HuN-XT-B: two representative PRRSV strains obtained in the present study)

Supplementary Table S1: Detailed information of PRRSV strains identified in this study and reference strains, including strain name, collection year, isolation region, genotype, and GenBank accession numbers.

**Strain**	**Isolation year**	**Isolation site**	**Lineage**	**GenBanK accession number**
JXA1	2006	Jiangxi, China	8.7 (HP-PRRSV strain)	EF112445
JXA1-P120	2009	Jiangxi, China	8.7 (HP-PRRSV strain)	KC422727
HuN	2007	Hunan, China	8.7 (HP-PRRSV strain)	EF517962
TJ	2006	Tianjin, China	8.7 (HP-PRRSV strain)	EU860248
FJZH	2015	Fujian, China	8.7 (HP-PRRSV strain)	KP998478
NJ-1106	2012	Shandong, China	8.7 (HP-PRRSV strain)	JX880029
HY21	2021	Hunan, China	8.7 (HP-PRRSV strain)	OL687155
CH-1a	1996	China	8.1 (Classical PRRSV strain)	AY032626
HH08	2011	China	8.1 (Classical PRRSV strain)	JX679179
HLJ	2017	Heilongjiang, China	8.1 (Classical PRRSV strain)	MH422083
SD53-1603	2016	Heilongjiang, China	1.8 (NADC30-like strain)	MH651744
NADC30	2017	Shandong, China	1.8 (NADC30-like strain)	MH500776
FJ203	2015	Fujian, China	1.8 (NADC30-like strain)	KP860909
CHsx1401	2014	Beijing, China	1.8 (NADC30-like strain)	KP861625
LNWK130	2017	Heilongjiang, China	1.5 (NADC34-like strain)	MG913987
NADC34	2014	USA	1.5 (NADC34-like strain)	MF326985
NCV-Anheal-1	2018	China	1.5 (NADC34-like strain)	MH370474
VR2332	1992	USA	5	U87392
BJ-4	1996	China	5	AF331831
QYYZ	2011	Guangdong, China	3	JQ308798
GM2	2011	China	3	JN662424
HuN-LD-A	2021	Loudi, Hunan, China	8.7 (HP-PRRSV strain)	OP219606
HuN-YY-C	2021	Yiyang, Hunan, China	8.7 (HP-PRRSV strain)	OP219607
HuN-SY-B	2021	Shaoyang, Hunan, China	8.7 (HP-PRRSV strain)	OP219608

HuN-ML-A2	2021	Miluo, Hunan, China	8.7 (HP-PRRSV strain)	OP219609
HuN-ZZ-B	2021	Zhuzhou, Hunan, China	8.7 (HP-PRRSV strain)	OP219610
HuN-ZJJ-A	2021	Zhangjiajie, Hunan, China	8.7 (HP-PRRSV strain)	OP219611
HuN-ZJJ-B	2021	Zhangjiajie, Hunan, China	8.7 (HP-PRRSV strain)	OP219612
HeB-HD-B	2021	Handan, Heibei, China	8.7 (HP-PRRSV strain)	OP219613
HeB-CD-B	2021	Chengde, Hebei, China	8.7 (HP-PRRSV strain)	OP219614
HeB-QHD-C	2021	Qinhuangdao, Hebei, China	8.7 (HP-PRRSV strain)	OP219615
HeB-SJZ-D	2021	Shijiazhuang, Hebei, China	8.7 (HP-PRRSV strain)	OP219616
HeB-TS-B	2021	Tangshan, Hebei, China	8.7 (HP-PRRSV strain)	OP219617
HuN-CD-B3	2021	Changde, Hunan, China	8.1 (Classical PRRSV strain)	OP219618
HuN-CD-B1	2021	Changde, Hunan, China	8.1 (Classical PRRSV strain)	OP219619
HuN-CD-B2	2021	Changde, Hunan, China	8.1 (Classical PRRSV strain)	OP219620
HeB-XT-B	2021	Xingtai, Hebei, China	1.8 (NADC30-like strain)	OP219621
HeB-ZJK-A	2021	Zhangjiakou, Hebei, China	1.8 (NADC30-like strain)	OP219622
HeB-BD-A	2021	Baoding, Hebei, China	1.8 (NADC30-like strain)	OP219623
HeB-HD-A	2021	Handan, Heibei, China	1.8 (NADC30-like strain)	OP219624
HeB-TS-A	2021	Tangshan, Hebei, China	1.8 (NADC30-like strain)	OP219625
HeB-SJZ-A	2021	Shijiazhuang, Hebei, China	1.8 (NADC30-like strain)	OP219626
HeB-SJZ-C	2021	Shijiazhuang, Hebei, China	1.8 (NADC30-like strain)	OP219627
HeB-XT-C	2021	Xingtai, Hebei, China	1.8 (NADC30-like strain)	OP219628
HeB-XT-A	2021	Xingtai, Hebei, China	1.8 (NADC30-like strain)	OP219629
HeB-SJZ-B	2021	Shijiazhuang, Hebei, China	1.8 (NADC30-like strain)	OP219630
HeB-QHD-A	2021	Qinhuangdao, Hebei, China	1.8 (NADC30-like strain)	OP219631
HeB-QHD-B	2021	Qinhuangdao, Hebei, China	1.8 (NADC30-like strain)	OP219632
HeB-BD-B	2021	Baoding, Hebei, China	1.8 (NADC30-like strain)	OP219633
HuN-YY-A	2021	Yiyang, Hunan, China	1.8 (NADC30-like strain)	OP219634
HuN-CS-A	2021	Changsha, Hunan, China	1.8 (NADC30-like strain)	OP219635
HuN-ZZ-A	2021	Zhuzhou, Hunan, China	1.8 (NADC30-like strain)	OP219636
HuN-YY-B	2021	Yiyang, Hunan, China	1.8 (NADC30-like strain)	OP219637

HuN-CS-B	2021	Changsha, Hunan, China	1.8 (NADC30-like strain)	OP219638
HuN-ML-A1	2021	Miluo, Hunan, China	1.8 (NADC30-like strain)	OP219639
HuN-LD-B	2021	Loudi, Hunan, China	1.8 (NADC30-like strain)	OP219640
HuN-XT-B	2021	Xiangtan, Hunan, China	1.8 (NADC30-like strain)	OP219641
HuN-XT-A	2021	Xiangtan, Hunan, China	1.8 (NADC30-like strain)	OP219642
HeB-XT-D	2021	Xingtai, Hebei, China	1.5 (NADC34-like strain)	OP219643
HeB-CD-A	2021	Chengde, Hebei, China	1.5 (NADC34-like strain)	OP219644
HuN-ZZ-C	2021	Zhuzhou, Hunan, China	3	OP219645
HuN-CD-B4	2021	Changde, Hunan, China	3	OP219646
HuN-SY-A	2021	Shaoyang, Hunan, China	3	OP219647
HuN-CD-A	2021	Changde, Hunan, China	3	OP219648

Supplemental Table S2. Comparison of GP5 amino acid sequences of sublineage 1.8 PRRSV strains identified in the present study.

Strains	Amino acid sites																												
	13	15	18	19	25	26	27	30	31	32-34	55	57-59	61	72	79	81	90	94	98	101	102	104	120	124	127	128	145	151	158	153	170
NADC30	Q	P	W	C	F	V	A	S	A	-ND	W	NEH	S	V	Y	A	T	I	T	Y	Y	K	L	A	L	A	L	R	P	R	E
HeB-BD-B	Q	P	W	C	F	A	A	N	A	SSN	W	DKR	S	A	Y	A	T	I	T	H	Y	G	L	A	L	A	L	K	P	K	E
HuN-YY-A	Q	P	W	C	F	A	V	N	A	SNN	W	DKK	D	V	Y	A	T	I	T	Y	Y	G	L	T	F	A	L	K	P	K	G
HeB-XT-A	Q	P	W	Y	F	A	A	N	A	SNN	W	GTR	S	V	Y	A	T	I	T	Y	F	K	L	A	L	A	L	K	P	R	G
HuN-CS-A	Q	L	W	C	F	V	A	N	A	MSN	W	DRH	S	A	Y	A	T	L	T	Y	Y	G	F	A	L	A	L	K	P	K	E
HuN-ZZ-A	Q	L	W	C	F	V	A	N	A	MSN	W	NKN	S	V	Y	A	T	I	T	Y	C	R	F	A	L	A	L	K	P	K	E
HuN-YY-B	Q	P	W	C	F	V	A	N	A	MSN	W	NKN	S	V	Y	A	T	I	T	Y	Y	R	F	A	L	A	L	K	P	K	E
HuN-CS-B	Q	L	W	C	F	V	A	N	A	MSN	W	NKN	S	V	Y	A	T	I	T	Y	Y	R	F	A	L	A	L	K	P	K	E
HuN-ML-A1	Q	L	W	C	F	V	A	N	A	MSN	W	NKN	S	V	Y	A	T	I	T	Y	Y	R	F	A	L	A	L	K	P	R	E
HuN-LD-B	Q	P	W	C	F	V	A	N	A	MSN	W	NKK	D	V	Y	A	T	A	I	Y	C	N	L	V	L	A	L	K	S	K	G
HuN-XT-A	Q	P	W	C	F	V	A	N	A	MSN	W	NKK	D	V	Y	A	T	A	I	Y	C	N	L	V	L	A	L	K	S	K	G
HuN-XT-B	Q	L	W	C	F	V	A	N	A	MSN	W	NSR	D	V	Y	A	T	A	T	Y	F	N	L	V	L	A	L	K	S	K	G
HeB-XT-B	Q	P	W	Y	L	A	A	T	A	SND	W	SEK	S	V	Y	A	M	V	T	Y	N	M	L	A	L	A	V	K	P	R	G
HeB-HD-A	Q	P	W	Y	L	A	A	T	V	SND	W	SEK	S	V	Y	A	M	V	T	Y	N	M	L	A	L	A	V	K	P	R	G

HeB-TS-A	Q	P	W	Y	L	A	A	T	V	SND	W	SEK	S	V	Y	A	M	V	T	Y	N	M	L	A	L	A	V	K	P	R	G
HeB-ZJK-A	Q	P	W	Y	L	A	A	T	A	SND	W	SEK	S	V	Y	A	M	V	T	Y	N	M	L	A	L	A	V	K	P	R	G
HeB-BD-A	Q	P	W	Y	L	A	A	T	A	SND	W	SEK	S	V	N	P	M	V	T	Y	N	M	L	A	L	A	V	K	P	R	G
HeB-SJZ-C	Q	P	W	Y	L	A	A	T	A	SND	R	SEK	S	V	Y	A	M	V	T	Y	N	M	L	A	L	A	V	K	P	R	G
HeB-SJZ-A	Q	P	W	Y	L	A	A	N	A	SND	G	SEK	S	V	Y	A	M	V	T	H	Y	K	L	A	L	A	V	K	P	R	G
HeB-XT-C	Q	P	W	Y	L	A	A	N	A	SND	W	SEK	S	V	Y	A	M	V	T	Y	Y	K	L	A	L	A	V	-	P	R	G
HeB-QHD-B	P	P	L	C	F	T	A	N	A	SNS	W	GKH	S	V	Y	A	T	I	T	Y	R	E	L	A	L	V	L	-	P	K	G
HeB-QHD-A	P	P	L	C	F	T	A	N	A	SNS	W	GKH	S	V	Y	A	T	I	T	Y	R	E	L	A	L	V	L	K	P	K	G
HeB-SJZ-B	P	P	L	C	F	T	A	N	A	SNS	W	GKH	S	V	Y	A	T	I	T	Y	R	E	L	A	L	V	L	K	P	K	G

Supplemental Table S3. Comparison of GP5 amino acid sequences of sublineage 8.7 PRRSV strains identified in the present study.

Strains	Amino acid sites												
	10	15	23	35	58	59	69	104	151	164	191	196	200
JXA1	C	L	F	N	Q	K	I	G	R	G	R	L	L
HuN-LD-A	C	L	F	N	Q	K	I	G	R	G	R	L	L
HuN-YY-C	C	L	F	N	Q	K	L	G	R	R	R	L	L
HuN-SY-B	C	P	F	N	Q	K	I	G	R	R	R	L	L
HuN-ML-A2	C	L	F	S	Q	K	I	G	R	R	R	L	L
HuN-ZZ-B	C	L	C	S	R	N	L	G	R	R	K	L	L
HeB-HD-B	C	L	S	N	Q	K	I	G	K	G	R	R	L
HuN-ZJJ-A	C	L	S	N	Q	K	I	G	K	G	R	R	L
HeB-CD-B	C	L	S	N	Q	Q	I	G	K	G	R	R	P
HeB-QHD-C	C	L	S	N	Q	K	I	A	K	G	R	R	P
HuN-ZJJ-B	C	L	S	N	Q	R	I	G	K	G	R	R	L
HeB-SJZ-D	F	P	F	N	Q	K	I	G	R	R	R	L	P
HeB-TS-B	F	P	F	N	Q	K	I	W	R	R	R	L	P
